# Light Trapping Enhancement in a Thin Film with 2D Conformal Periodic Hexagonal Arrays

**DOI:** 10.1186/s11671-015-0988-y

**Published:** 2015-07-08

**Authors:** Xi Yang, Suqiong Zhou, Dan Wang, Jian He, Jun Zhou, Xiaofeng Li, Pingqi Gao, Jichun Ye

**Affiliations:** Ningbo Institute of Material Technology and Engineering, Chinese Academy of Sciences, Ningbo, 315201 China; Institute of Photonics, Faculty of Science, Ningbo University, Ningbo, 315211 Zhejiang China; College of Physics, Optoelectronics and Energy & Collaborative Innovation Center of Suzhou Nano Science and Technology, Soochow University, Suzhou, 215006 China; Key Lab of Advanced Optical Manufacturing Technologies of Jiangsu Province & Key Lab of Modern Optical Technologies of Education Ministry of China, Soochow University, Suzhou, 215006 China

**Keywords:** Light trapping, Photonic crystals, Nanospheres, Plasmonics, Rear reflectors

## Abstract

Applying a periodic light trapping array is an effective method to improve the optical properties in thin-film solar cells. In this work, we experimentally and theoretically investigate the light trapping properties of two-dimensional periodic hexagonal arrays in the framework of a conformal amorphous silicon film. Compared with the planar reference, the double-sided conformal periodic structures with all feature periodicities of sub-wavelength (300 nm), mid-wavelength (640 nm), and infrared wavelength (2300 nm) show significant broadband absorption enhancements under wide angles. The films with an optimum periodicity of 300 nm exhibit outstanding antireflection and excellent trade-off between light scattering performance and parasitic absorption loss. The average absorption of the optimum structure with a thickness of 160 nm is 64.8 %, which is much larger than the planar counterpart of 38.5 %. The methodology applied in this work can be generalized to rational design of other types of high-performance thin-film photovoltaic devices based on a broad range of materials.

**Electronic supplementary material:**

The online version of this article (doi:10.1186/s11671-015-0988-y) contains supplementary material, which is available to authorized users.

## Background

To reduce the usage of materials and the production cost of modules, a substantial amount of researches have been focused on lightweight and mechanically flexible thin-film solar cells (TFSCs). Due to the significantly reduced thickness, the commercially used light trapping structures based on surface texturing and antireflection coating are insufficient for the broadband light absorption of TFSCs [1]. Alternatively, nanostructures, such as nanobowls, nanoshells, nanocones, and zigzags on the front side [2–8] and metal nanoparticles (or reflectors) on the back side [9–14], have been extensively studied to achieve advanced light trapping through guiding the incident light into the TFSCs or/and the induced localized surface plasmonics and strongly light scattering for the increase of the optical path length.

In many cases, it has been proved that periodic photonic nanostructures outperform the random counterparts in light trapping. For example, Cui et al. achieved a solar cell with a short-circuit current density (*J*_sc_) of 17.1 mA/cm^2^ and an initial efficiency of 10.9 % using a 250-nm-thick amorphous silicon absorber on the periodic nanocavity substrate [15]. Atwater et al. designed and fabricated periodic and random arrays of nanoscatters in the back contact of a hydrogenated amorphous silicon (a-Si:H) solar cell, and the efficiency of the periodic arrays with a pitch size of 400 nm came to be 9.60 % which was superior to the random one of 9.35 % and planar one of 6.32 % [16]. It is worth noting that light absorption can be enhanced by the combination of top periodic nanogratings for effective antireflection and the bottom periodic nanogratings for efficient light scattering [17, 18]. The double-sided arrangement of the film could strengthen the coupling of the incident light into waveguide modes and store the incident energy in a localized surface plasmon mode. Cui et al. demonstrated nanodome solar cells with a 280-nm-thick a-Si:H layer, which combined many nanophotonic effects to both efficiently reduce reflection and enhance absorption and could absorb 94 % of the light with wavelengths of 400–800 nm [19, 20]. Tan et al. demonstrated the self-assembled silver nanoparticle (Ag NP)-based plasmonic back reflector (BR) in a-Si:H solar cells with a *J*_sc_ as high as 15.1 mA/cm^2^ [21]. Iftiquar et al. investigated a-Si:H solar cells deposited on pyramidally multitextured substrates and achieved a great improvement in the *J*_sc_ by 1.3 mA/cm^2^ due to a further reduction in reflection loss of the incident light [22, 23]. Fan et al. fabricated thin-film a-Si:H solar cells based on i-cone substrates with 0.5 aspect ratio and demonstrated the highest energy conversion efficiency, which is 34 % higher than that of the flat reference device [24]. Nevertheless, the optimized double-sided structure depends on the periodicity with different unabsorbed solar spectrum profiles for light trapping and the absorbing capabilities of the film at different wavelengths. The optimization of the periodicity is a trade-off between a few factors, including surface reflection reduction, light scattering, and parasitic absorption. Small feature sizes exhibit strong reflection reduction and low scattering, while large feature sizes show strong scattering and even higher order scattering modes with comparatively parasitic absorption loss. Therefore, a complete understanding of the precise relationship between the periodicity and photoabsorption enhancement within the absorbing layer should be considered.

In our work, two-dimensional (2D) periodic gratings with feature sizes of sub-wavelength (300 nm), mid-wavelength (640 nm), and infrared wavelength (2300 nm) are fabricated using a hexagonal close-packed colloidal polystyrene (PS) sphere method. The Ag film and the ultrathin a-Si film are then conformally deposited on the periodic substrates in order to evaluate the optical characteristics of the periodic structures. Without any antireflection coating, the conformal ultrathin film (160 nm in thicknesses) with a sub-wavelength periodicity of 300 nm shows a broadband absorption enhancement with a 68 % increase over the planar reference (from 38.5 to 64.8 %) throughout the whole wavelength range. Furthermore, we simulate the optical absorption of such conformal structures by varying their structural periodicity to determine the optimum case that gives rise to maximum solar absorption. The insights in this work open up a new guidance for achieving efficient photoabsorption of thin films.

## Methods

### Fabrication of Periodic Hexagonal Arrays

**Fig. 1:**
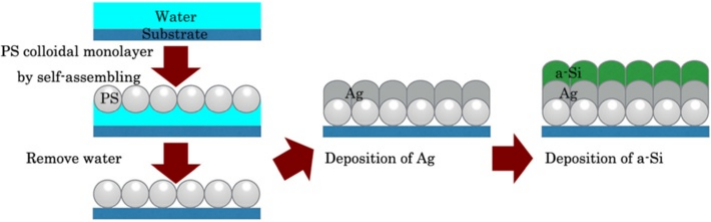
Fabrication. Schematic of the conformal periodic hexagonal array fabrication

Figure 1 outlines the procedure for the fabrication of 2D conformal periodic hexagonal arrays. Aqueous colloidal PS spheres with different diameters were synthesized by emulsifier-free emulsion polymerization [25]. The glass substrates were treated in a HNO_3_ solution with a concentration of 50 % at 60 °C to enhance hydrophilicity and then were subsequently cleaned using acetone, ethanol, and distilled water in an ultrasonic bath. About 10 ml colloidal PS spheres were injected into the water with an injection speed of 1.5 μl/s to form a Langmuir-Blodgett (LB) film in a hexagonal configuration at the air/water interface [26]. By draining the water, the LB film was transferred to the glass substrate, which had been previously immersed in the water. The LB film of PS spheres adhered tightly to the glass substrate after annealing at 80 °C for 10 min. A silver (Ag) film and an a-Si film were successively and conformably deposited through sputtering onto the hexagonally arranged PS LB film at a deposition rate of ~7 and 3.3 Å/s, respectively.

### SEM and SPM Measurements

**Fig. 2:**
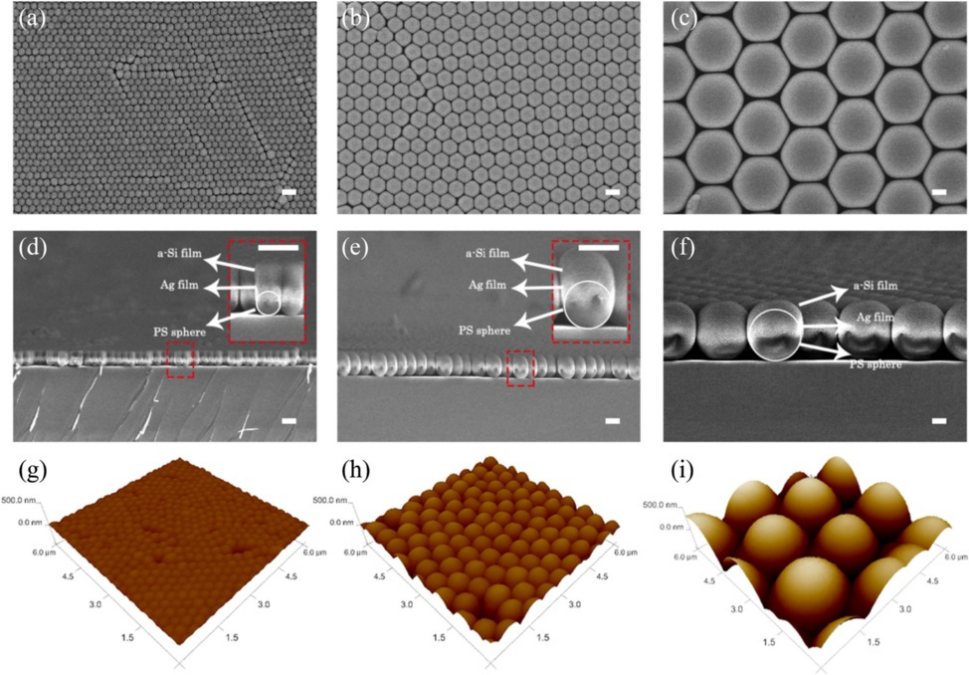
SEM and AFM characterization results. The top-view SEM images of the conformal hexagonal structures with different periodicities: 300 nm (**a**), 600 nm (**b**), and 2 μm (**c**). The corresponding cross-sectional SEM image of the same configurations (**d**–**f**). All the *scale bars* are 500 nm. The tapping-mode SPM images of the surface topography of the conformal a-Si film with periodicities: 300 nm (**g**), 600 nm (**h**), and 2 μm (**i**)

The scanning electron microscopy (SEM) characterization and the tapping-mode scanning probe microscope (SPM) measurement were conducted using a Hitachi S-4800 SEM and a Veeco 3100 SPM, respectively. Figure 2 shows the top-view (a–c) and cross-sectional SEM images (d–f), and SPM scans (g–i) of the surface topography of the conformal a-Si films with three types of periodicities.

### Spectrophotometric Measurements

The optical characterization of the samples over the range of wavelength from 300 to 900 nm was performed using a Lamda950 UV-VIS-VIR spectrometer (PerkinElmer) equipped with a 150-mm-diameter integrating sphere to account for total light (diffuse and specular). Incident light entered the sphere through a small port and illuminated the sample mounted at the rear of the sphere. The reflected light was scattered uniformly by the interior sphere wall. A silicon detector mounted at the back of the sphere produced a photocurrent from all the reflected photons. The reflection (*R*) could be collected and detected. Assuming the transmission (*T*) was zero because of the silver backscattering reflector (Ag-BSR), the total absorption (*A*) could be calculated as *A* = 1 − *R*.

### Light Absorption Enhancement Theoretical Modeling

**Fig. 3:**
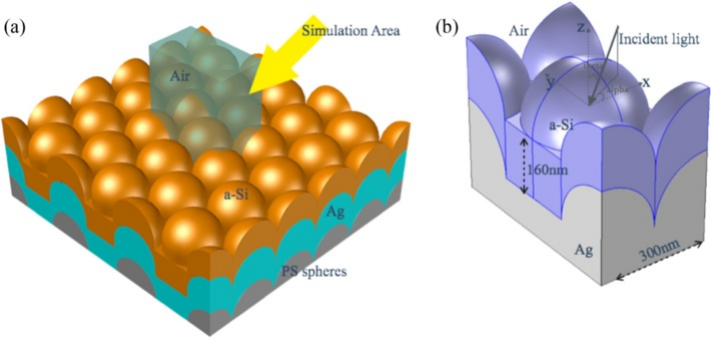
Realistic photovoltaic systems. **a** Schematic drawing of a typical 3D optical model used in the simulations (not to scale); the Ag film (*green*) and a-Si film (*orange*) are strictly deposited on the hexagonal periodic nanospheres (*gray*). **b** The unit cells of the 300-nm periodic light trapping structure with 160-nm-thick a-Si. The PS spheres in **b** are not indicated because the Ag film is thick enough to prevent light from transmitting

To further characterize the light trapping effect related to the surface topography and the feature sizes of the structures, the optical behaviors are examined through numerical simulation. Figure 3a depicts the three-dimensional (3D) model of the conformal structures with periodic hexagonal configurations investigated in our simulation. For convenience, we have assumed that the Ag and a-Si films are strictly deposited on the close-packed periodic hexagonal PS nanospheres without any defects, and the configurations are rigorously conformal from bottom to top. The unit cell of the optimized structure is shown in Fig. 3b. The thickness of the a-Si layer is 160 nm, and the periodicity of the hexagonal array is 300 nm, as can be verified from Fig. 2. The incident light is a plane wave with a polar angle, *θ*, and an azimuthal angle, *φ*. The light wavelength, *λ*, is chosen from 300 to 900 nm to match the a-Si absorption band. The layer thicknesses are taken from the cross sections of SEM and SPM measurements in Fig. 2. The optical index of a-Si is based on the ellipsometry measurement (Additional file 1), and the optical parameter of Ag is based on the Palik data [27]. Both the transverse electric (TE) and transverse magnetic (TM) polarizations are simulated. The simulations are performed by finite element method within a unit configuration surrounded by the periodic boundaries along the lateral direction [28]. The perfectly matched layers (PML) are implemented along the incident direction to prevent interference effect. The detailed energy flux distribution inside the structures is calculated under the solar spectrum at air mass 1.5 [29]. The absorption and the reflection at each wavelength are calculated with an interval of 10 nm.

## Results and Discussion

### Experimental Results

**Fig. 4:**
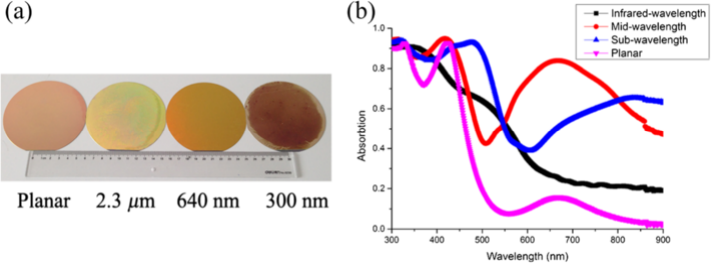
Photographs and the absorption curves of the four samples. **a** Photographs of 4-in. wafer-sized samples with infrared wavelength (2300 nm), mid-wavelength (640 nm), and sub-wavelength (300 nm) periodicities as well as a planar reference. **b** The measured absorption spectra of all four samples. The thickness of the a-Si layer and Ag layer is 160 and 200 nm, respectively

Figure 4a shows photographs of four a-Si samples, i.e., planar setup and periodically arrayed systems under three configurations (*P* = 300, 640, and 2300 nm) under an identical film thickness (160 nm). It is shown that (1) the planar film is mirror-like and highly reflective, (2) the sample with an infrared wavelength periodicity of 2300 nm reflects less, and (3) the sample based on mid-wavelength (sub-wavelength) looks green (dark brown), revealing the strong optical absorption capability. The spectrometer is used to quantitatively characterize the absorption of these samples over a broad range of wavelength (*λ*) from 300 to 900 nm, which covers most of the solar spectrum. Figure 4b shows the measured absorption spectra of the planar structure and hexagonal arrays with three periodicities. Compared with the planar reference, the films with the periodic arrays demonstrate a significantly improved absorption in the longer wavelength range of 600 nm < *λ* < 900 nm, where Ag-BSR and the parasitic absorption of Ag are dominating. Within 350 nm < *λ* < 600 nm, the absorption peaks are ascribed to the Fabry-Perot (FP) cavity modes induced by the stacking structure of air/a-Si/Ag. The resonance wavelength of the FP cavity mode has a redshift with decreasing *P*. In the shorter wavelength range of *λ* < 350 nm, there is no significant enhancement of the absorption over the planar reference as the absorption is inherently strong for the a-Si material. It is speculated that the average absorption of the film over the whole wavelength increases when the periodicity migrates from the infrared wavelength range to the sub-wavelength range, which is mainly due to the enhancement of the scattering caused by the Ag-BSR. The experimental result also certifies that the samples with *P* = 300 nm have an average absorption of 60 %, which is nearly twice that of the planar sample, showing the most outstanding light trapping capability among the three periodic structures.

**Fig. 5:**
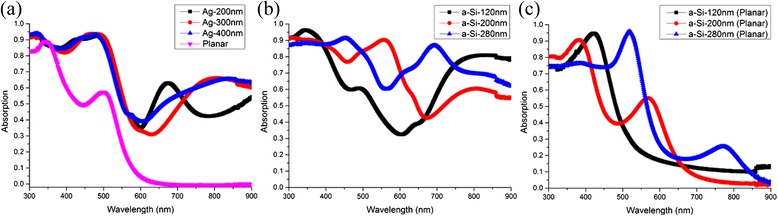
Absorption spectra. **a** Absorption spectra of 300-nm periodic samples with a Ag layer of 200-, 300-, and 400-nm thicknesses; the planar film with the Ag layer of 200-nm thickness is used as a reference, and the thickness of the a-Si layer in all four samples is 160 nm. **b** Absorption spectra of 300-nm periodic samples with an a-Si layer of 120-, 200-, and 280-nm thicknesses. **c** The absorption spectra of the three planar samples are used as the references relative to the periodic ones in **b**; the thickness of Ag in **b** and **c** is fixed at 200 nm

To further explore the benefits brought by the conformal hexagonal configuration, the absorption properties of the structures with different thicknesses of the Ag and a-Si films are investigated in detail. All these studies are based on the structures with *P* = 300 nm, which leads to the highest light trapping performance according to our experiments. It is worth noting that the surface morphology flattens out when the deposited films (Ag and a-Si) are getting thicker. Figure 5a shows the absorption spectra of periodic samples with a top a-Si layer of 160 nm and a bottom Ag layer of 200, 300, and 400 nm, respectively. The absorptions of all three samples in the short wavelength ranges (300–550 nm) are similar, and they start to be different at a longer wavelength range of *λ* > 550 nm mainly due to the different surface topographies induced by the different thicknesses of the Ag layer. It is worth noting that the absorption peaks are redshifted with increasing thickness of the Ag film. The top surface topography would change the incident angles of the light that reaches the bottom and affect light absorption, although the exact effect on light confinement at long wavelength is difficult to predict. Figure 5b depicts the absorption spectra of samples with a Ag layer of 200 nm but with an a-Si film of various thicknesses. With the increasing thickness of the a-Si film, the average absorption at the wavelength range of 300 nm < *λ* < 600 nm increases and the absorption peaks arising from the FP cavity modes are redshifted. At the wavelength range of *λ* > 600 nm, the light trapping of the periodic structures is distinctively superior to that of the planar reference as shown in Fig. 5c. Compared with the planar one, the average absorption of the optimum structure with an a-Si layer of 280-nm thickness and a Ag layer of 200-nm thickness is boosted by 71.6 %, from 45.1 to 77.4 % over the whole wavelength due to the presence of the periodic configuration.

### Modeling Results

**Fig. 6:**
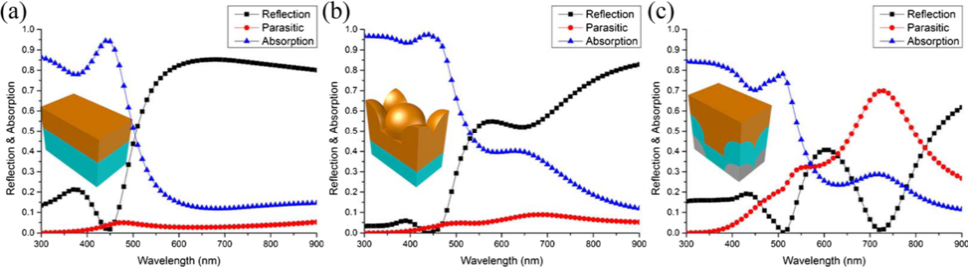
Simulated reflection and the absorption spectra of three samples. **a** The planar, **b** the top-only, and **c** the bottom-only structures. The *insets* are the sketches of the relevant structures

For a deeper insight into the antireflection of the top periodic surface and the light trapping of the bottom periodic grating, we compare the performance of optimized “top-only” and “bottom-only” grating structures to that of a planar thin-film structure with the same equivalent thickness. Firstly, the top surface of the planar reference in Fig. 6a is replaced by the hexagonally ordered arrays with a sub-wavelength periodicity of 300 nm in Fig. 6b. Compared to the planar reference, the absorption enhancement of the periodic structures is due to the significant antireflection of the top hexagonally arranged surface in the shorter wavelength range of *λ* < 550 nm, while in the longer wavelength range of *λ* > 550 nm, the small enhancement is observed because more incident light is trapped into the a-Si layer between the top structures and the planar bottom reflector. Secondly, the bottom layer of the planar reference (Fig. 6a) is substituted by a hexagonal configuration with a sub-wavelength periodicity of 300 nm (Fig. 6c) to characterize the light trapping effect of the bottom surface configurations. It is observed that with the periodic arrays on the bottom, a slightly absorption enhancement is achieved in the range of *λ* < 550 nm except for a narrow band centered at *λ* ~ 450 nm, where the strong FP cavity excited from the planar references is destroyed. In the wavelength range of *λ* > 550 nm, the absorption is rapidly enhanced due to the light scatting by the bottom hexagonal configuration. The periodic back Ag layers couple the light into the waveguide modes that propagate in the plane of the a-Si layer. The peak absorption at the wavelength around 720 nm is attributed to the surface plasmon polaritons (SPPs) although the SPP waves have higher losses due to the parasitic absorption in the metal film.

**Fig. 7:**
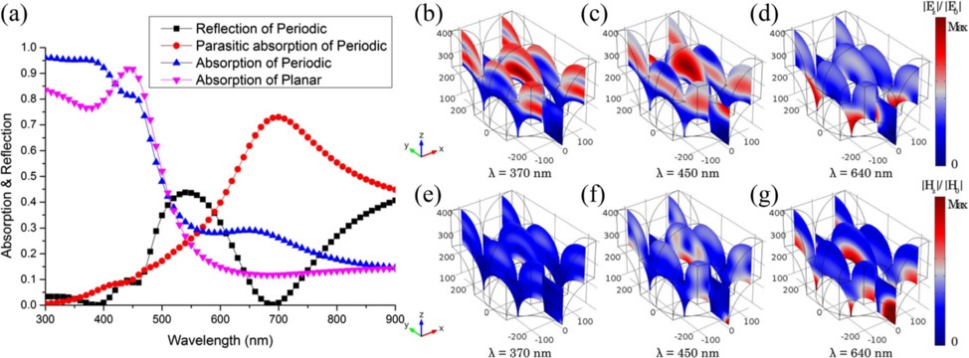
Distributions of optical absorption. **a** The simulated absorption spectra of the 300-nm period structure and the planar reference over the wavelength range from 300 to 900 nm. Distributions of normalized electric field for TE polarization at the wavelengths **b** *λ* = 370 nm, **c** *λ* = 450 nm, and **d** *λ* = 640 nm. Distributions of normalized magnetic field for TM polarization at absorption peak wavelengths **e** *λ* = 370 nm, **f** *λ* = 450 nm, and **g** *λ* = 640 nm

**Fig. 8:**
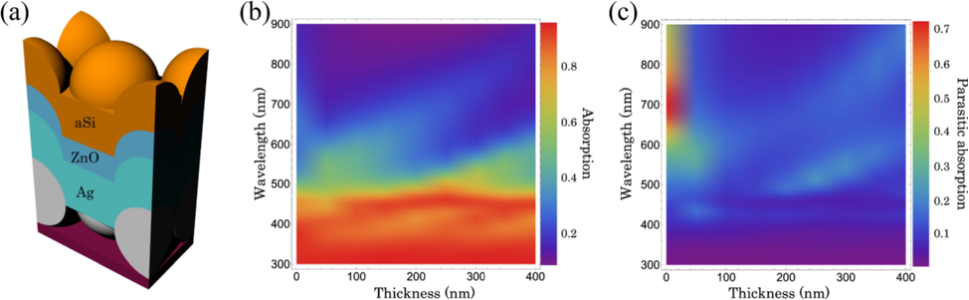
Absorption of the optimum structure with a ZnO layer. **a** The schematic drawing of a typical 3D optical model with a ZnO layer between the a-Si and Ag layers; the thickness of the a-Si and Ag layers is 160 and 200 nm, respectively. Simulated absorption of the a-Si layer (**b**) and parasitic absorption of the Ag layer (**c**) as a function of the illumination wavelength and thickness of the ZnO layer

Taking into account the significant difference in the structural requirements for antireflection and light trapping, the optical performance of the double-sided periodic conformal structure with both the top and the bottom hexagonally periodic arrays is simulated. As shown in Fig. 7a, the absorption of the hexagonal configurations is obviously enhanced over the whole wavelength compared with the planar reference. The absorption patterns at typical wavelengths in Figs. 6g and 7b well illustrate the absorption inside the periodic structures and satisfactorily verify the previous observation and explanation. For TE polarization, three Bloch modes are clearly observed at absorption peak wavelengths, namely, *λ* = 370, 450, and 640 nm, whose electric field distributions are shown in Fig. 7b–d, respectively. For TM polarization, the situation is different, and we plot the magnetic field distributions at the absorption peak wavelengths in Fig. 7e–g, respectively. The first mechanism of absorption enhancement is related to cavity resonance and can be seen in Fig. 7b obviously. The interference within the thin film leads to a resonant FP cavity effect, depending on the thickness of the layer. Another resonance, which is shown in Fig. 7c, is introduced by adding the periodic patterns, whose mode strength is related to the parameters of the Ag back patterns. The second mechanism is related to SPPs that can be excited only by TM polarization, which can be inferred from the Hz field observation in Fig. 7g. The third mechanism will be associated with the coupling into waveguide modes, which can be found in the middle of the active layer. Because of these mechanisms, the absorption spectra are enhanced and broadened significantly for both TE and TM polarizations although the SPP waves still have higher losses due to the parasitic absorption in the metal film as shown from the red curve in Fig. 7a. To characterize the loss of parasitic absorption, a thin layer of ZnO film is placed between the active layer and Ag-BSR as a dielectric spacer to reduce metallic loss. Figure 8 shows the schematic of the optimized structures (a), the absorption of the active layer (b), and the parasitic loss of the metallic layer (c) as a function of wavelength and thickness (*W*) of the ZnO layer. When *W* < 50 nm, the metallic parasitic loss accompanied by the excitation of SPPs in the longer wavelength range is still high. With the increasing *W*, the grating at the bottom of the active layer becomes far from the metal surface and the parasitic absorption of the metal is reduced while the scattering effect of the back surface morphology also slackens. When *W* = 100 nm, the Ag-BSR induces no significant parasitic loss and keeps good light scattering properties. In this case, the ZnO layer not only acts as a diffusion buffer layer between a-Si and Ag but also optically separates the a-Si waveguide modes from the lossy SPP modes on the metallic interface.

**Fig. 9:**
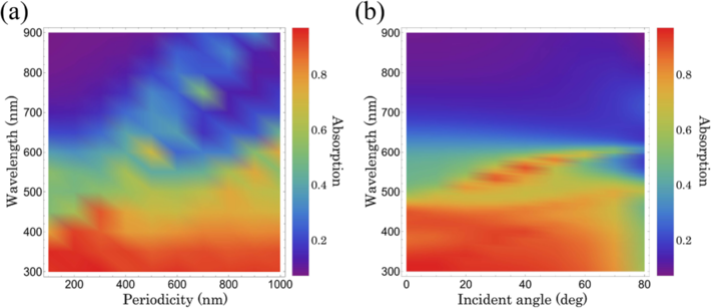
Dependence of absorption on the periodicity and incident angle. **a** Calculated absorption of a-Si under normal incident light versus varied periodicity and wavelength. The thickness of a-Si is fixed at 160 nm. **b** Calculated absorption of a-Si versus varied incident angle and wavelength. The thickness of a-Si is 160 nm, and the periodicity is 300 nm

Furthermore, we theoretically study the active film on the conformal ordered arrays with periodicity ranging from sub-wavelength to 2× typical wavelengths (100–1000 nm) on the front surface of the 100-nm-thick ZnO layer and optimize it to maximize photoabsorption within a given thickness of 160 nm. As shown in Fig. 9a, with increasing *P*, the absorption peaks are redshifted, degrading the average absorption due to the low reflection reduction and the parasitic absorption; however, when *P* < 300 nm, a weak scattering leads to a higher optical loss at a longer wavelength range and hinders the absorption improvement. The structure with *P* = 300 nm has the highest average absorption after the trade-off between the light scattering and the parasitic absorption [30]. Finally, the angular dependence of the light absorption is also interesting to consider. The properties of diffraction gratings are naturally dependent on the incident angle. The behavior under oblique illumination conditions is an important part of the performance investigation of periodic structures. We consider only a single wavelength of the extended spectrum that reaches the back side, and we neglect the fact that the diffraction efficiencies will also change with the incident angle. The polar angle of the diffracted orders *θ*_0_ can be found from Eq. (1) [17].

1 $$ { \sin}^2\left({\theta}_0\right)=2{\left(\frac{n_i}{n_0} \sin \left(\theta \right) \cos \left(\varphi \right)+\frac{m\lambda }{n_0P}\right)}^2, $$

where *m* is an integer that denotes the diffraction order, and *n*_*i*_ and *n*_*o*_ are the refractive index of the medium of incidence and of the outgoing wave, respectively. The angular performance of the designed system is examined briefly in Fig. 9b, where the optimized structures have stable absorption spectra from 0° to 60°. The reduction in absorption at higher angles of incidence is the escape of diffraction orders that are no longer totally internally reflected within the patterned film.

## Conclusions

We experimentally achieved a large-area 2D periodic hexagonal structure with a broadband light trapping effect. Without any antireflection coating, the ordered arrays with a sub-wavelength periodicity of 300 nm demonstrated more than three times absorption enhancement over the planar reference in the long wavelength range. The numerical simulation verified that the absorption enhancement was achieved by combining the merits of both top- and bottom-side conformal periodic structures. For the lights in the shorter wavelength range, which could not penetrate the absorber and reach the bottom surface, the top-sided periodic structures increase the absorption by reducing the top-sided reflectance. For the lights in the long wavelength range wherein the film is not thick enough to completely absorb, the bottom-sided periodic structures couple the lights into the waveguide modes or excite the surface plasmon polaritons, which could both result in absorption enhancement. To avoid the parasitic absorption caused by the Ag reflector, a 100-nm ZnO layer is introduced between the absorption layer and rear Ag reflector. The optimum structures have stable absorption spectra with little angle dependence. This large-area fabrication of the conformal hexagonal structures provided a realistic strategy in efficiency improvement for the next-generation thin-film solar cells.

## Additional file

Additional file 1:
**Additional material.** A document showing the optical index of a-Si and measurements of the diameters of the PS spheres.

## Abbreviations

Ag-BSR: silver backscattering reflector
FP: Fabry-Perot
LB: Langmuir-Blodgett
PML: perfectly matched layers
PS: polystyrene
SEM: scanning electron microscopy
SPM: scanning probe microscope
SPPs: surface plasmon polaritons
TE: transverse electric
TFSCs: thin-film solar cells
TM: transverse magnetic

## References

[1] Green MA (2002). Lambertian light trapping in textured solar cells and light-emitting diodes: analytical solutions. Prog Photovoltaics.

[2] Grandidier J, Callahan DM, Munday JN, Atwater HA (2011). Light absorption enhancement in thin-film solar cells using whispering gallery modes in dielectric nanospheres. Adv Mater.

[3] Gao P, Wang H, Sun Z, Han W, Li J, Ye J (2013). Efficient light trapping in low aspect-ratio honeycomb nanobowl surface texturing for crystalline silicon solar cell applications. Appl Phys Lett.

[4] Brongersma ML, Cui Y, Fan SH (2014). Light management for photovoltaics using high-index nanostructures. Nat Mater.

[5] Zhu J, Yu ZF, Burkhard GF, Hsu CM, Connor ST, Xu YQ (2009). Optical absorption enhancement in amorphous silicon nanowire and nanocone arrays. Nano Lett.

[6] Yang Z, Shang A, Zhan Y, Zhang C, Li X (2013). Ultra-broadband performance enhancement of thin-film amorphous silicon solar cells with conformal zig-zag configuration. Opt Lett.

[7] Sheng J, Fan K, Wang D, Han C, Fang J, Gao P (2014). Improvement of the SiO passivation layer for high-efficiency Si/PEDOT:PSS heterojunction solar cells. ACS Appl Mater Interfaces.

[8] Zhang C, Li X, Shang A, Wu S, Zhan Y, Yang Z (2014). Design of dual-diameter nanoholes for efficient solar-light harvesting. Nanoscale Res Lett.

[9] Ferry VE, Verschuuren MA, Li HBBT, Schropp REI, Atwater HA, Polman A (2009). Improved red-response in thin film a-Si:H solar cells with soft-imprinted plasmonic back reflectors. Appl Phys Lett.

[10] Ferry VE, Verschuuren MA, Li HBT, Verhagen E, Walters RJ, Schropp REI (2010). Light trapping in ultrathin plasmonic solar cells. Opt Express.

[11] Bhattacharya J, Chakravarty N, Pattnaik S, Slafer WD, Biswas R, Dalal VL (2011). A photonic-plasmonic structure for enhancing light absorption in thin film solar cells. Appl Phys Lett.

[12] Paetzold UW, Moulin E, Michaelis D, Bottler W, Wachter C, Hagemann V (2011). Plasmonic reflection grating back contacts for microcrystalline silicon solar cells. Appl Phys Lett.

[13] Paetzold UW, Moulin E, Pieters BE, Carius R, Rau U (2011). Design of nanostructured plasmonic back contacts for thin-film silicon solar cells. Opt Express.

[14] Zhang YA, Stokes N, Jia B, Fan SH, Gu M (2014). Towards ultra-thin plasmonic silicon wafer solar cells with minimized efficiency loss. Sci Rep-Uk.

[15] Battaglia C, Hsu CM, Soderstrom K, Escarre J, Haug FJ, Charriere M (2012). Light trapping in solar cells: can periodic beat random?. ACS Nano.

[16] Ferry VE, Verschuuren MA, van Lare MC, Schropp REI, Atwater HA, Polman A (2011). Optimized spatial correlations for broadband light trapping nanopatterns in high efficiency ultrathin film a-Si:H solar cells. Nano Lett.

[17] Gjessing J, Sudbo AS, Marstein ES (2011). Comparison of periodic light-trapping structures in thin crystalline silicon solar cells. J Appl Phys.

[18] Eisenlohr J, Benick J, Peters M, Blasi B, Goldschmidt JC, Hermle M (2014). Hexagonal sphere gratings for enhanced light trapping in crystalline silicon solar cells. Opt Express.

[19] Hsu C-M, Battaglia C, Pahud C, Ruan Z, Haug F-J, Fan S (2012). High-efficiency amorphous silicon solar cell on a periodic nanocone back reflector. Adv Energy Mater.

[20] Zhu J, Hsu CM, Yu Z, Fan S, Cui Y (2010). Nanodome solar cells with efficient light management and self-cleaning. Nano Lett.

[21] Tan H, Santbergen R, Smets AHM, Zeman M (2012). Plasmonic light trapping in thin-film silicon solar cells with improved self-assembled silver nanoparticles. Nano Lett.

[22] Iftiquar SM, Jung J, Shin C, Park H, Park J, Jung J (2015). Light management for enhanced efficiency of textured n–i–p type amorphous silicon solar cell. Sol Energy Mater Sol Cells.

[23] Iftiquar SM, Jung J, Park H, Cho J, Shin C, Park J (2015). Effect of light trapping in an amorphous silicon solar cell. Thin Solid Films.

[24] Lin Q, Leung S, Lu L, Chen X, Chen Z, Tang HN (2014). Inverted nanocone-based thin film photovoltaics with omnidirectionally enhanced performance. ACS Nano.

[25] Zhang J, Chen Z, Wang Z, Zhang W, Ming N (2003). Preparation of monodisperse polystyrene spheres in aqueous alcohol system. Mater Lett.

[26] Gao P, He J, Zhou S, Yang X, Li S, Sheng J, et al. Large-area nanosphere self-assembly by a micro-propulsive injection method for high throughput periodic surface nanotexturing. Nano Letters. 2015. doi:10.1021/acs.nanolett.5B01202. 10.1021/acs.nanolett.5b01202 26039258

[27] Palik ED. Handbook of optical constants of solids. Orlando: Academic; 1985.

[28] Comsol Multiphysics. [http://www.comsol.com/]

[29] Hylton NP, Li X, Giannini V, Lee K, Ekins-Daukes NJ, Loo J (2013). Loss mitigation in plasmonic solar cells: aluminium nanoparticles for broadband photocurrent enhancements in GaAs photodiodes. Sci Rep.

[30] ASTM. Reference solar spectral irradiance: AM 1.5 spectra, http://rredc.nrel.gov/solar/spectra/am1.5/

