# Supplementary material for: Light Trapping Enhancement in a Thin Film with 2D Conformal Periodic Hexagonal Arrays
Source: Nanoscale Res Lett. 2015 Jul 8;10:284. doi: 10.1186/s11671-015-0988-y (PMC4495099; doi:10.1186/s11671-015-0988-y)
Supplement: Additional file 1: — Additional material. A document showing the optical index of a-Si and measurements of the diameters of the PS spheres. [file 11671_2015_988_MOESM1_ESM.pdf]

Additional material

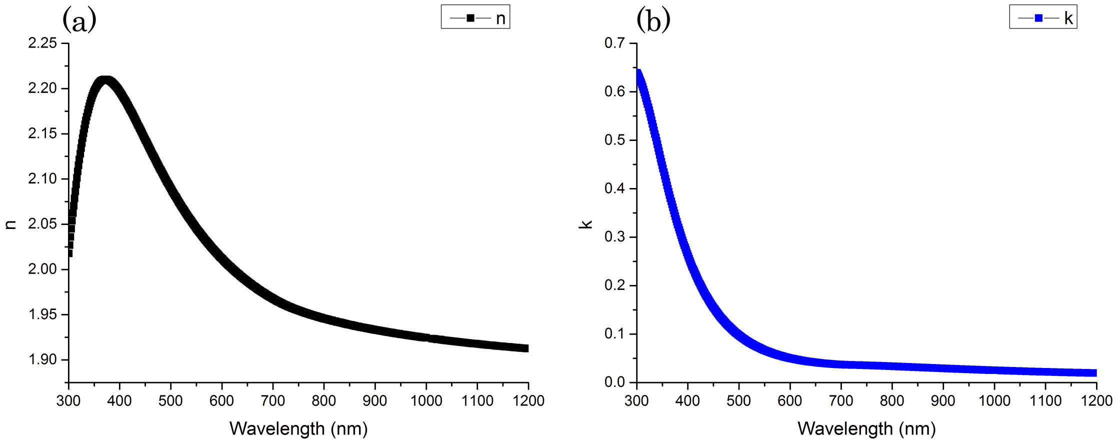

The optical index of a-Si based on the ellipsometry measurement. (a) n, (b)k.

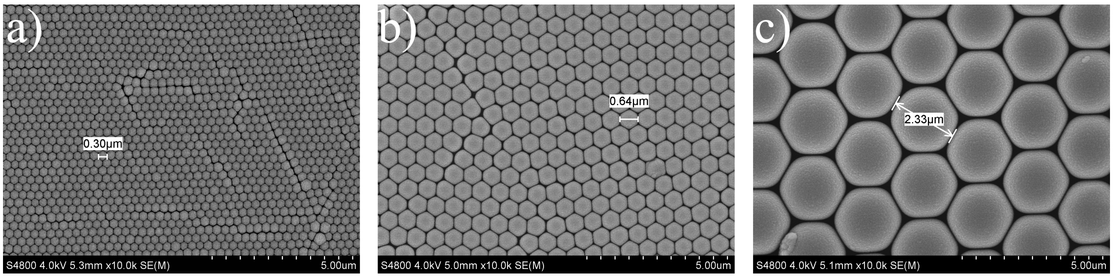

Measurements of the diameters of the PS spheres with three types of sizes. a) sub-wavelength (300 nm), b) mid-wavelength (640 nm) and c) infrared-wavelength (2300 nm)
